# Supplementary material for: Deletion of wfs1 Impairs Oligodendrocyte Precursor Cells Dorsal Distribution and Myelination Through the wfs1-hmgcs1 Axis in Zebrafish
Source: Biology (Basel). 2026 Aug 16;15(16):1402. doi: 10.3390/biology15161402 (PMC13510151; doi:10.3390/biology15161402)
Supplement: Supplementary file 1 [file biology-15-01402-s001.zip › File S1-Transcriptome sequencing data and differentially expressed genes (DEGs) in wfs1-- zebrafish at 5 dpf/2.KEGG/wfs1KOvsWT/ALL/src/dre04910.html]

dre04910


- dre:100330617

- Up regulated genes

100330617\_1(1.54741913113035)

- dre:559001

- Down regulated genes

559001\_1(-1.11683329469951)

- dre:100001260

- Up regulated genes

100001260\_1(3.49284240013245)

- Down regulated genes

100001260(-10.2224161507053)
- dre:406459

- Up regulated genes

406459(2.32535160749006)

- dre:192322

- Up regulated genes

336121(1.42904963424916)
- dre:569706

- Up regulated genes

569706\_1(4.25727678218935)

- dre:559403

- Up regulated genes

559403\_1(1.4336766768898)

- dre:393444

- Up regulated genes

393444(1.27911287872533)

- dre:79380

- Up regulated genes

79380(9.11141682868863)

- Down regulated genes

79380\_1(-1.05168581152728)

- dre:58100

- Down regulated genes

58100\_1(-4.1286149197908)

- dre:406473

- Up regulated genes

406473(3.55746093515464)

- dre:100000252

- Up regulated genes

100000252\_3(1.94434154854557) 100000252(1.09163460881879)

- Down regulated genes

100000252\_1(-1.7718817168434)

- dre:406339

- Down regulated genes

406339\_1(-1.38680349052238)
- dre:321224

- Up regulated genes

321224(1.93576834540418)
- dre:751668

- Up regulated genes

751668(2.48606367672347)

- dre:556277

- Up regulated genes

556277(2.56614816386233)

Close
